# Supplementary material for: Development and validation of LC-MS/MS method for imatinib and norimatinib monitoring by finger-prick DBS in gastrointestinal stromal tumor patients
Source: PLoS One. 2019 Nov 19;14(11):e0225225. doi: 10.1371/journal.pone.0225225 (PMC6863526; doi:10.1371/journal.pone.0225225)
Supplement: Attachment — Submitted filename: Response to Reviewers.docx [file pone.0225225.s001.docx]

Reviewer #1:

Minor editing for English grammar and usage.

*The manuscript has been revised for English grammar.*

Line 104 should have a reference.

*As properly suggested by Reviewer, a reference has been added to line 104 related to the advantages of DBS in TDM practice.*

Which model Millipore was used to produce water in line 135.

*The Millipore model used was Milli-Q^®^ IQ 7000. This information has been added to the manuscript (Line 130).*

Working solutions and calibrators are claimed to be made "from A to L", however only ten working solutions and calibrators were identified (Lines 150, 152, 161 & 163).

*The Reviewer is perfectly right since the Italian alphabet was mistakenly used to name the working solutions (thus from A to L we have only 10 letters). The manuscript has been revised according to the English alphabet (thus the working solutions are now named from A to J).*

Line 345 states that the clinical protocol was designed to collect blood "without anticoagulant", however lines 319 & 320 state that in the clinical protocol, venous blood was collected with K-EDTA, an anti-coagulant. Was a separate vacutainer collected for the correlation between finger prick and venous collection?

*The Reviewer is right since the description of samples collection (Line 318-328) was erroneously reported. In fact, 1 mL of venous blood was collected without anticoagulant using a syringe and directly transferred into 1.5 mL tube. The blood was immediately spotted with a Gilson pipette on a piece of Whatman ET31CHR filter paper to make Control – DBS (the spots were 20 µL in volume). This step needed to be fast to avoid blood coagulation inside the tubes. The chapter has been properly corrected.*

Line 505, indicate "LCCC" stands for Lin’s Concordance Correlation Coefficient.

*The LCCC abbreviation was introduced at the first mention of Lin’s concordance correlation coefficient (Line 373). Anyway it could be easier for the reader to make explicit what LCCC stands for also in Line 505 since it is an uncommon abbreviation.*

Typo on Line 542 "LC-M/MS"

*We thank the reviewer for finding this typo that has been corrected.*

Reviewer #2:

I think the article is well written in a straightforward manner, thus easy to follow by the readers. The validation experiments done by the authors are comprehensive and sufficient. There are only a few minor revisions/addendum:

L152, 159-163: There is some error in listing of quality control concentrations, the final concentrations do not add up from the working concentrations. It is suggested to keep the order of concentrations consistent, best listed in an ascending manner.

*We thank the reviewer for finding the error in quality control concentrations reported in Line 152 that have been substituted with the correct ones (120, 25,5 µg/mL).*

L301-304: The HPLC parameters changed from the method development (L186-189) to the plasma sample analysis. Please discuss why it has changed and the effects it brought. Normally, the method validated should be kept the same in GLP conditions.

*The LC-MS/MS method applied for the quantification of IMA and norIMA in patients plasma sample was already in use in our laboratory (it is currently under revision by Journal of Pharmaceutical and Biomedical Analysis with the following title: “A New High-Performance Liquid Chromatography-Tandem Mass Spectrometry Method for Therapeutic Drug Monitoring of Imatinib and Norimatinib in GIST patients“). It was developed and validated according to FDA and EMA guidelines before the development of the assay for the quantification of DBS samples and thus it was used as REFERENCE method during the cross validation study (Line 294-295). DBS samples were analysed with the proposed method (COMPARANTOR method) while plasma samples were analysed with the reference method: the respective results were finally compared applying statistical analyses such as Passing-Bablok regression, Bland-Altman method, Lin’s concordance correlation coefficient and FDA/EMA requirements.*

L449: change “follow” to “follows”

*Done.*
